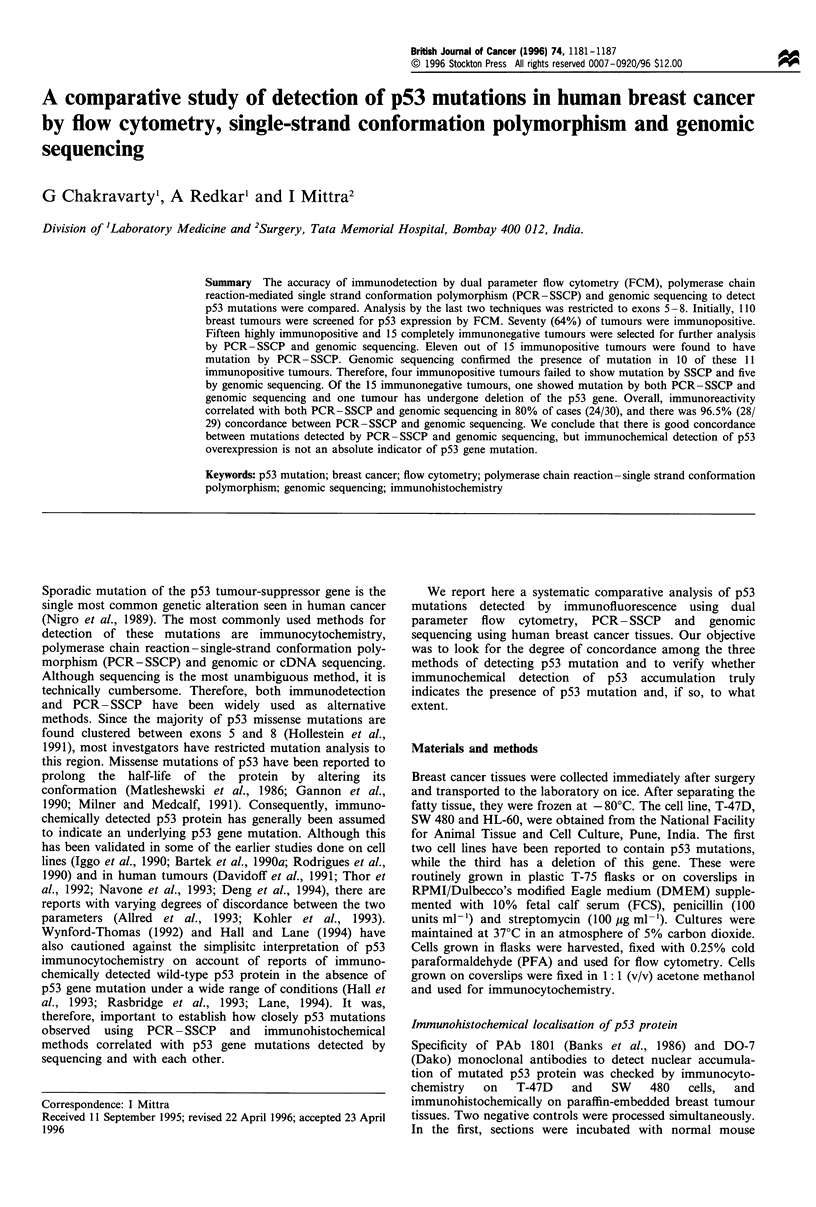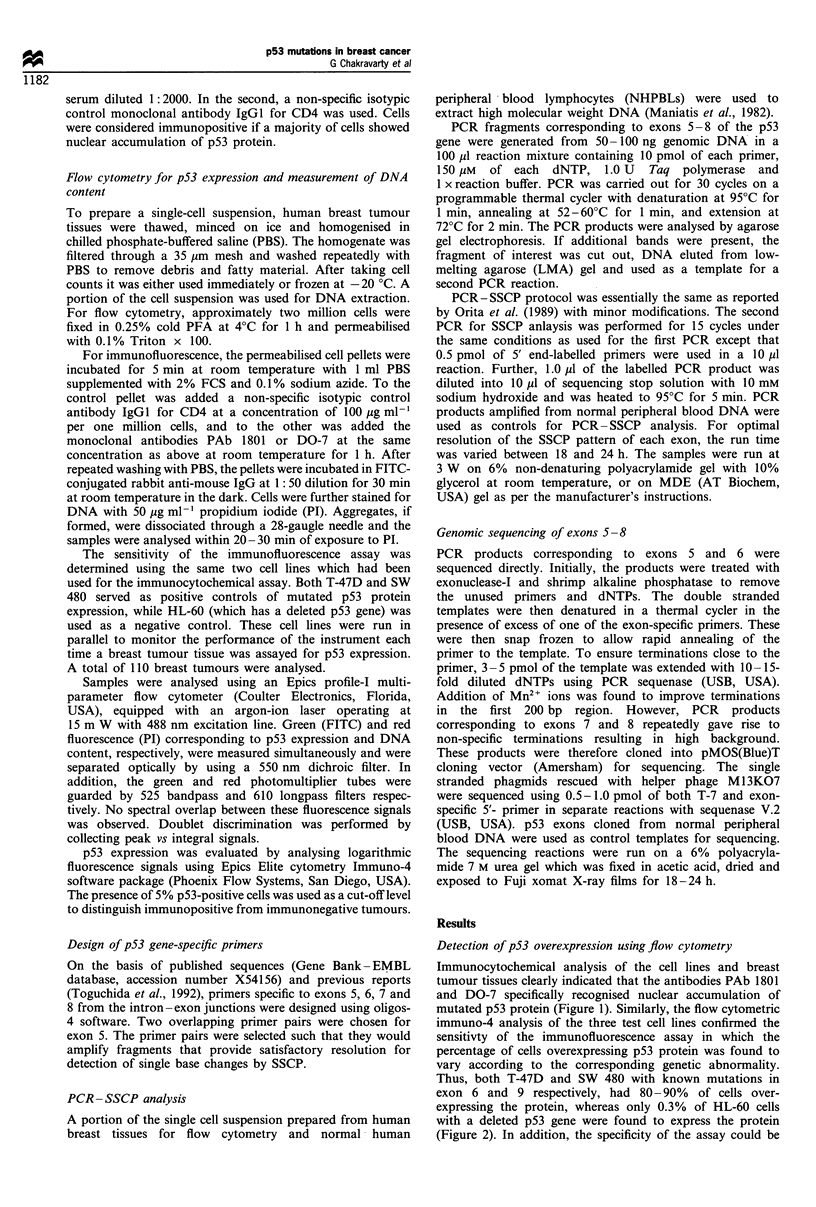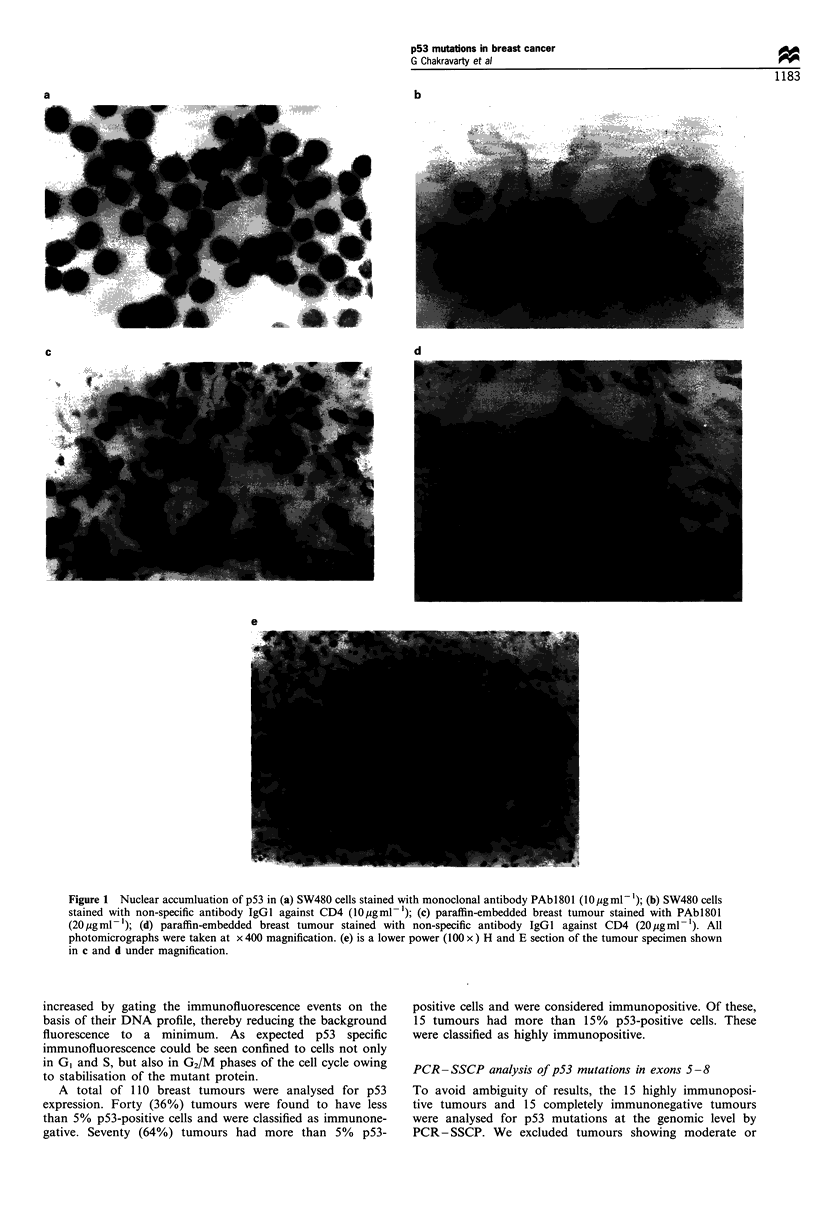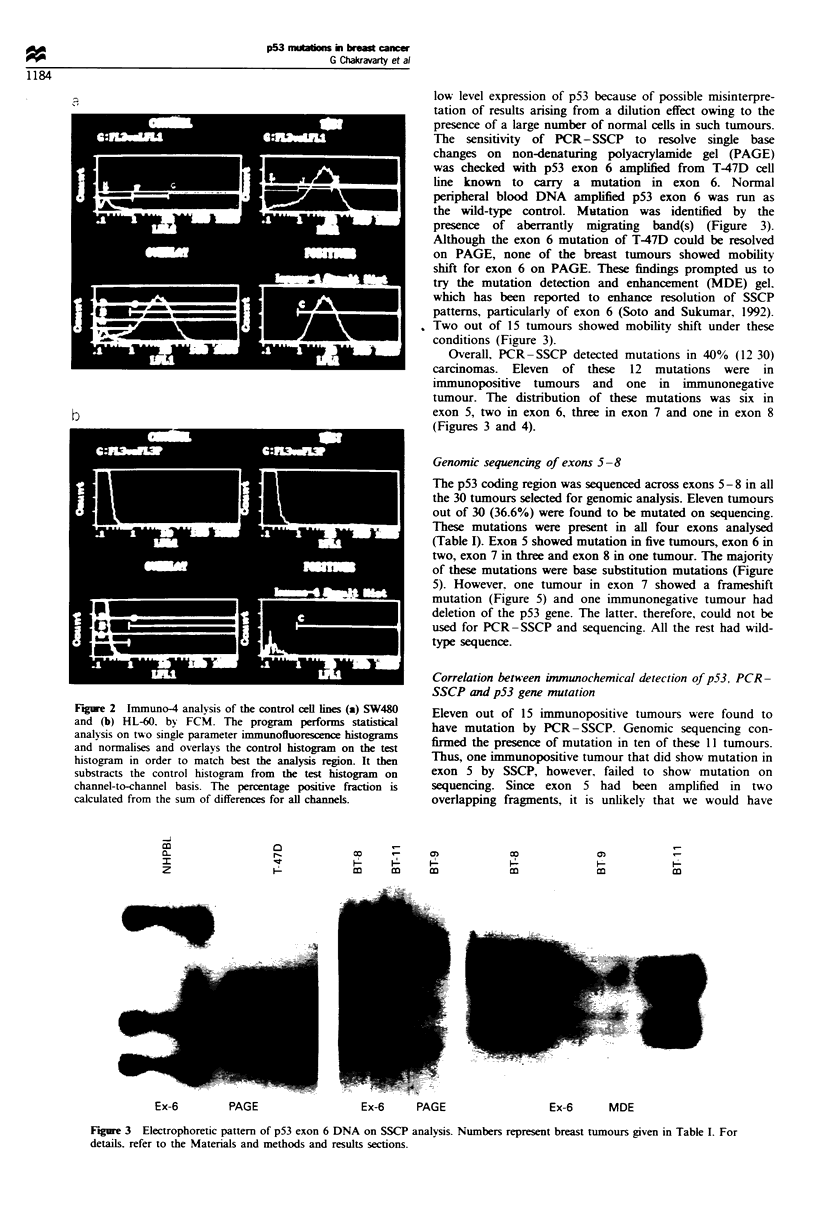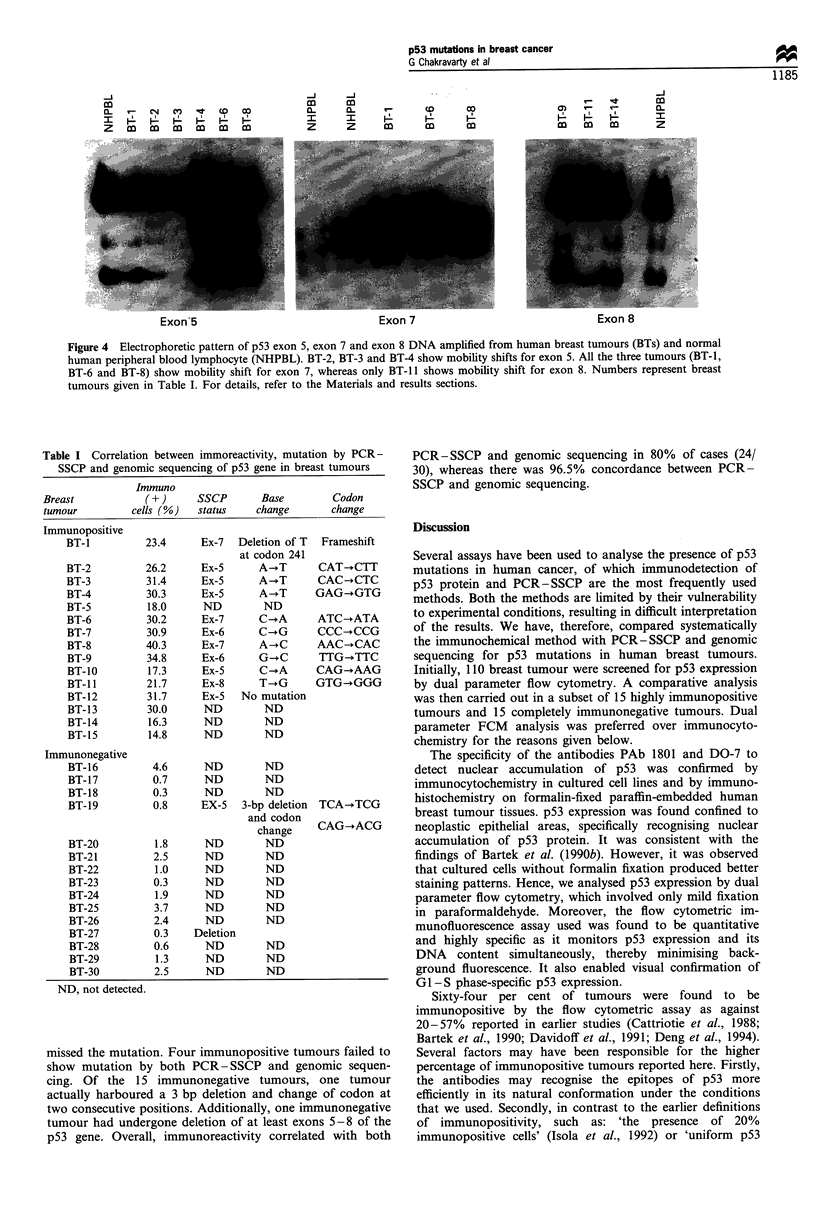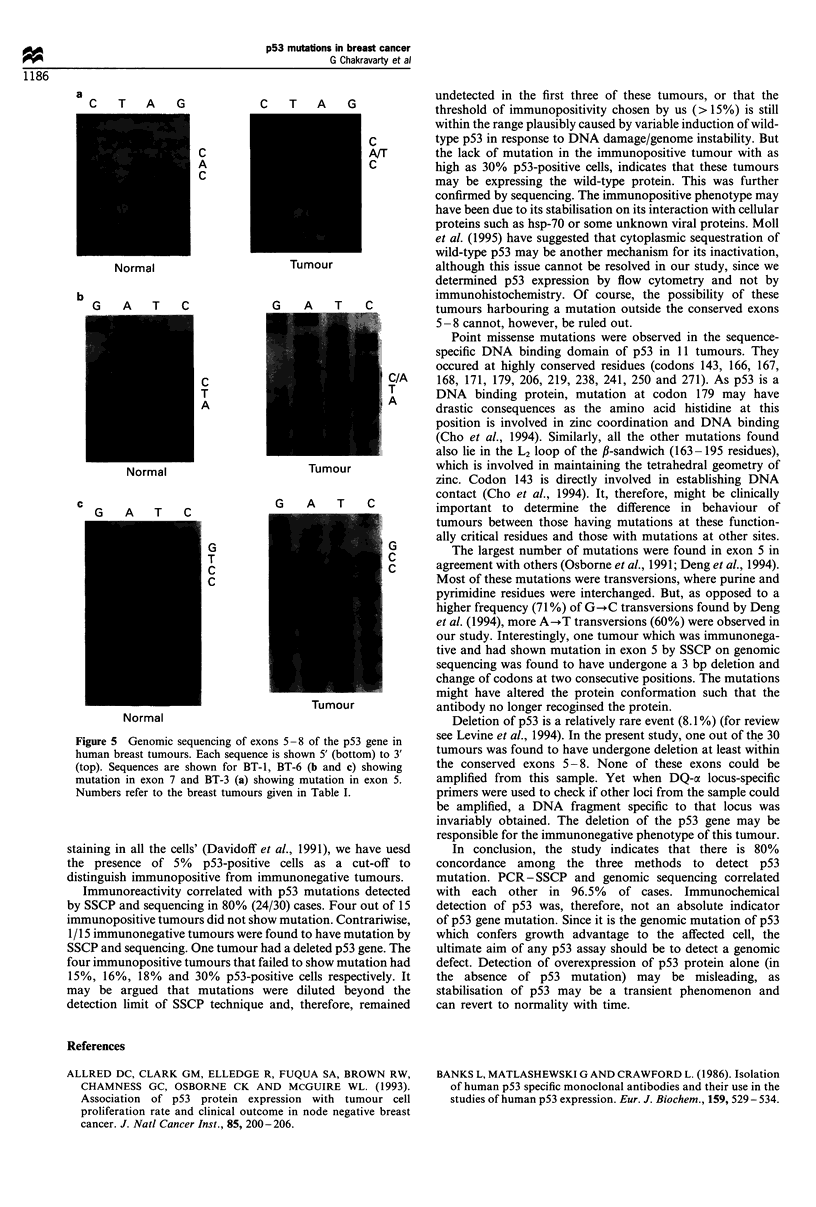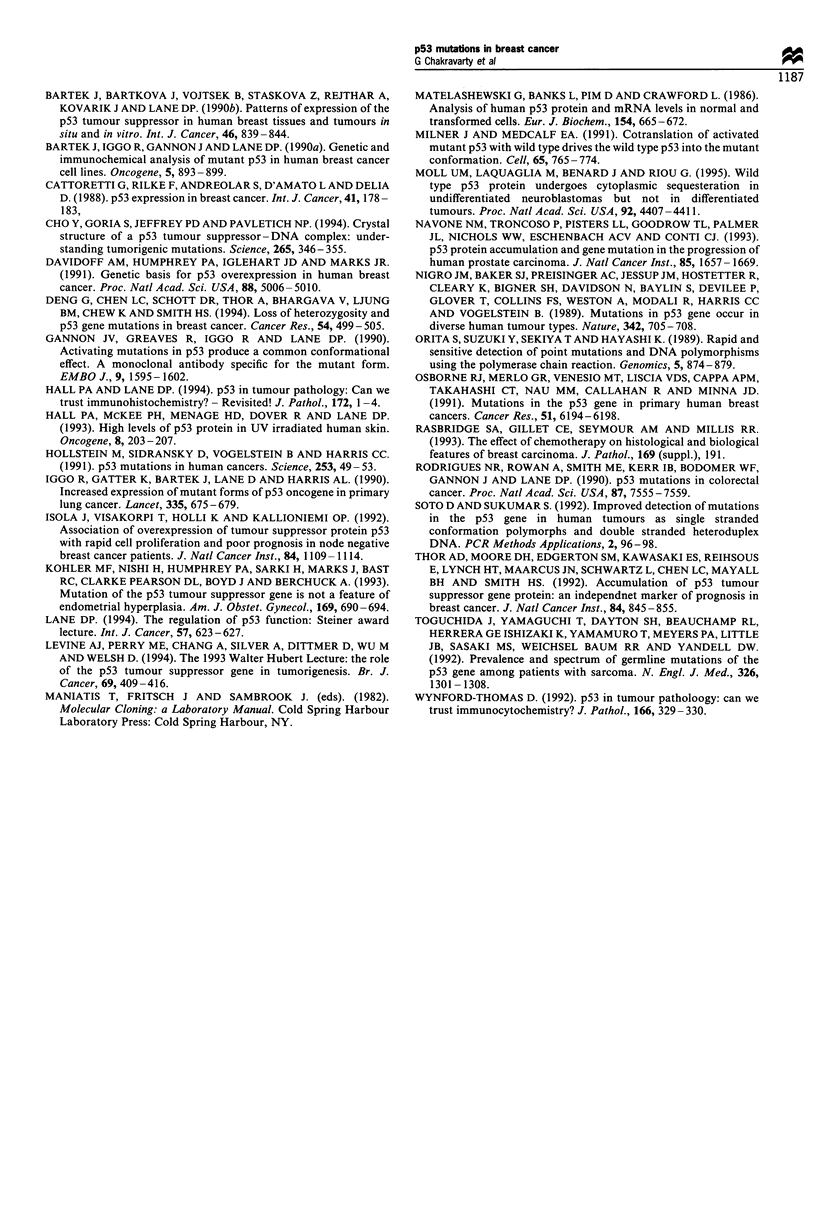# A comparative study of detection of p53 mutations in human breast cancer by flow cytometry, single-strand conformation polymorphism and genomic sequencing.

**DOI:** 10.1038/bjc.1996.514

**Published:** 1996-10

**Authors:** G. Chakravarty, A. Redkar, I. Mittra

**Affiliations:** Division of Laboratory Medicine, Tata Memorial Hospital, Bombay, India.

## Abstract

**Images:**


British Journal of Cancer (1996) 74, 1181-1187
© 1996 Stockton Press All rights reserved 0007-0920/96 $12.00

### A comparative study of detection of p53 mutations in human breast cancer by flow cytometry, single-strand conformation polymorphism and genomic sequencing

G Chakravarty[1], A Redkar[1] and I Mittra[2]

*Division of [1]Laboratory Medicine and [2]Surgery, Tata Memorial Hospital, Bombay 400 012, India.*

**Summary** The accuracy of immunodetection by dual parameter flow cytometry (FCM), polymerase chain reaction-mediated single strand conformation polymorphism (PCR–SSCP) and genomic sequencing to detect p53 mutations were compared. Analysis by the last two techniques was restricted to exons 5–8. Initially, 110 breast tumours were screened for p53 expression by FCM. Seventy (64%) of tumours were immunopositive. Fifteen highly immunopositive and 15 completely immunonegative tumours were selected for further analysis by PCR–SSCP and genomic sequencing. Eleven out of 15 immunopositive tumours were found to have mutation by PCR–SSCP. Genomic sequencing confirmed the presence of mutation in 10 of these 11 immunopositive tumours. Therefore, four immunopositive tumours failed to show mutation by SSCP and five by genomic sequencing. Of the 15 immunonegative tumours, one showed mutation by both PCR–SSCP and genomic sequencing and one tumour has undergone deletion of the p53 gene. Overall, immunoreactivity correlated with both PCR–SSCP and genomic sequencing in 80% of cases (24/30), and there was 96.5% (28/29) concordance between PCR–SSCP and genomic sequencing. We conclude that there is good concordance between mutations detected by PCR–SSCP and genomic sequencing, but immunochemical detection of p53 overexpression is not an absolute indicator of p53 gene mutation.

**Keywords:** p53 mutation; breast cancer; flow cytometry; polymerase chain reaction–single strand conformation polymorphism; genomic sequencing; immunohistochemistry

Sporadic mutation of the p53 tumour-suppressor gene is the single most common genetic alteration seen in human cancer (Nigro *et al.*, 1989). The most commonly used methods for detection of these mutations are immunocytochemistry, polymerase chain reaction–single-strand conformation polymorphism (PCR–SSCP) and genomic or cDNA sequencing. Although sequencing is the most unambiguous method, it is technically cumbersome. Therefore, both immunodetection and PCR–SSCP have been widely used as alternative methods. Since the majority of p53 missense mutations are found clustered between exons 5 and 8 (Hollestein *et al.*, 1991), most investgators have restricted mutation analysis to this region. Missense mutations of p53 have been reported to prolong the half-life of the protein by altering its conformation (Matleshewski *et al.*, 1986; Gannon *et al.*, 1990; Milner and Medcalf, 1991). Consequently, immunochemically detected p53 protein has generally been assumed to indicate an underlying p53 gene mutation. Although this has been validated in some of the earlier studies done on cell lines (Iggo *et al.*, 1990; Bartek *et al.*, 1990*a*; Rodrigues *et al.*, 1990) and in human tumours (Davidoff *et al.*, 1991; Thor *et al.*, 1992; Navone *et al.*, 1993; Deng *et al.*, 1994), there are reports with varying degrees of discordance between the two parameters (Allred *et al.*, 1993; Kohler *et al.*, 1993). Wynford-Thomas (1992) and Hall and Lane (1994) have also cautioned against the simplisitc interpretation of p53 immunocytochemistry on account of reports of immunochemically detected wild-type p53 protein in the absence of p53 gene mutation under a wide range of conditions (Hall *et al.*, 1993; Rasbridge *et al.*, 1993; Lane, 1994). It was, therefore, important to establish how closely p53 mutations observed using PCR–SSCP and immunohistochemical methods correlated with p53 gene mutations detected by sequencing and with each other.

Correspondence: I Mittra
Received 11 September 1995; revised 22 April 1996; accepted 23 April 1996

We report here a systematic comparative analysis of p53 mutations detected by immunofluorescence using dual parameter flow cytometry, PCR–SSCP and genomic sequencing using human breast cancer tissues. Our objective was to look for the degree of concordance among the three methods of detecting p53 mutation and to verify whether immunochemical detection of p53 accumulation truly indicates the presence of p53 mutation and, if so, to what extent.

### Materials and methods

Breast cancer tissues were collected immediately after surgery and transported to the laboratory on ice. After separating the fatty tissue, they were frozen at −80°C. The cell line, T-47D, SW 480 and HL-60, were obtained from the National Facility for Animal Tissue and Cell Culture, Pune, India. The first two cell lines have been reported to contain p53 mutations, while the third has a deletion of this gene. These were routinely grown in plastic T-75 flasks or on coverslips in RPMI/Dulbecco's modified Eagle medium (DMEM) supplemented with 10% fetal calf serum (FCS), penicillin (100 units $ml^{-1}$) and streptomycin (100 $\mu g\ ml^{-1}$). Cultures were maintained at 37°C in an atmosphere of 5% carbon dioxide. Cells grown in flasks were harvested, fixed with 0.25% cold paraformaldehyde (PFA) and used for flow cytometry. Cells grown on coverslips were fixed in 1 : 1 (v/v) acetone methanol and used for immunocytochemistry.

### *Immunohistochemical localisation of p53 protein*

Specificity of PAb 1801 (Banks *et al.*, 1986) and DO-7 (Dako) monoclonal antibodies to detect nuclear accumulation of mutated p53 protein was checked by immunocytochemistry on T-47D and SW 480 cells, and immunohistochemically on paraffin-embedded breast tumour tissues. Two negative controls were processed simultaneously. In the first, sections were incubated with normal mouse serum diluted 1 : 2000. In the second, a non-specific isotypic control monoclonal antibody IgG1 for CD4 was used. Cells were considered immunopositive if a majority of cells showed nuclear accumulation of p53 protein.

*Flow cytometry for p53 expression and measurement of DNA content*

To prepare a single-cell suspension, human breast tumour tissues were thawed, minced on ice and homogenised in chilled phosphate-buffered saline (PBS). The homogenate was filtered through a 35 $\mu$m mesh and washed repeatedly with PBS to remove debris and fatty material. After taking cell counts it was either used immediately or frozen at −20 °C. A portion of the cell suspension was used for DNA extraction. For flow cytometry, approximately two million cells were fixed in 0.25% cold PFA at 4°C for 1 h and permeabilised with 0.1% Triton × 100.

For immunofluorescence, the permeabilised cell pellets were incubated for 5 min at room temperature with 1 ml PBS supplemented with 2% FCS and 0.1% sodium azide. To the control pellet was added a non-specific isotypic control antibody IgG1 for CD4 at a concentration of 100 $\mu g\ ml^{-1}$ per one million cells, and to the other was added the monoclonal antibodies PAb 1801 or DO-7 at the same concentration as above at room temperature for 1 h. After repeated washing with PBS, the pellets were incubated in FITC-conjugated rabbit anti-mouse IgG at 1 : 50 dilution for 30 min at room temperature in the dark. Cells were further stained for DNA with 50 $\mu g\ ml^{-1}$ propidium iodide (PI). Aggregates, if formed, were dissociated through a 28-gaugle needle and the samples were analysed within 20−30 min of exposure to PI.

The sensitivity of the immunofluorescence assay was determined using the same two cell lines which had been used for the immunocytochemical assay. Both T-47D and SW 480 served as positive controls of mutated p53 protein expression, while HL-60 (which has a deleted p53 gene) was used as a negative control. These cell lines were run in parallel to monitor the performance of the instrument each time a breast tumour tissue was assayed for p53 expression. A total of 110 breast tumours were analysed.

Samples were analysed using an Epics profile-I multi-parameter flow cytometer (Coulter Electronics, Florida, USA), equipped with an argon-ion laser operating at 15 m W with 488 nm excitation line. Green (FITC) and red fluorescence (PI) corresponding to p53 expression and DNA content, respectively, were measured simultaneously and were separated optically by using a 550 nm dichroic filter. In addition, the green and red photomultiplier tubes were guarded by 525 bandpass and 610 longpass filters respectively. No spectral overlap between these fluorescence signals was observed. Doublet discrimination was performed by collecting peak *vs* integral signals.

p53 expression was evaluated by analysing logarithmic fluorescence signals using Epics Elite cytometry Immuno-4 software package (Phoenix Flow Systems, San Diego, USA). The presence of 5% p53-positive cells was used as a cut-off level to distinguish immunopositive from immunonegative tumours.

*Design of p53 gene-specific primers*

On the basis of published sequences (Gene Bank−EMBL database, accession number X54156) and previous reports (Toguchida *et al*., 1992), primers specific to exons 5, 6, 7 and 8 from the intron−exon junctions were designed using oligos-4 software. Two overlapping primer pairs were chosen for exon 5. The primer pairs were selected such that they would amplify fragments that provide satisfactory resolution for detection of single base changes by SSCP.

*PCR−SSCP analysis*

A portion of the single cell suspension prepared from human breast tissues for flow cytometry and normal human peripheral blood lymphocytes (NHPBLs) were used to extract high molecular weight DNA (Maniatis *et al*., 1982).

PCR fragments corresponding to exons 5−8 of the p53 gene were generated from 50−100 ng genomic DNA in a 100 $\mu$l reaction mixture containing 10 pmol of each primer, 150 $\mu$M of each dNTP, 1.0 U *Taq* polymerase and 1 × reaction buffer. PCR was carried out for 30 cycles on a programmable thermal cycler with denaturation at 95°C for 1 min, annealing at 52−60°C for 1 min, and extension at 72°C for 2 min. The PCR products were analysed by agarose gel electrophoresis. If additional bands were present, the fragment of interest was cut out, DNA eluted from low-melting agarose (LMA) gel and used as a template for a second PCR reaction.

PCR−SSCP protocol was essentially the same as reported by Orita *et al*. (1989) with minor modifications. The second PCR for SSCP anlaysis was performed for 15 cycles under the same conditions as used for the first PCR except that 0.5 pmol of 5′ end-labelled primers were used in a 10 $\mu$l reaction. Further, 1.0 $\mu$l of the labelled PCR product was diluted into 10 $\mu$l of sequencing stop solution with 10 mM sodium hydroxide and was heated to 95°C for 5 min. PCR products amplified from normal peripheral blood DNA were used as controls for PCR−SSCP analysis. For optimal resolution of the SSCP pattern of each exon, the run time was varied between 18 and 24 h. The samples were run at 3 W on 6% non-denaturing polyacrylamide gel with 10% glycerol at room temperature, or on MDE (AT Biochem, USA) gel as per the manufacturer's instructions.

*Genomic sequencing of exons 5−8*

PCR products corresponding to exons 5 and 6 were sequenced directly. Initially, the products were treated with exonuclease-I and shrimp alkaline phosphatase to remove the unused primers and dNTPs. The double stranded templates were then denatured in a thermal cycler in the presence of excess of one of the exon-specific primers. These were then snap frozen to allow rapid annealing of the primer to the template. To ensure terminations close to the primer, 3−5 pmol of the template was extended with 10−15-fold diluted dNTPs using PCR sequenase (USB, USA). Addition of $Mn^{2+}$ ions was found to improve terminations in the first 200 bp region. However, PCR products corresponding to exons 7 and 8 repeatedly gave rise to non-specific terminations resulting in high background. These products were therefore cloned into pMOS(Blue)T cloning vector (Amersham) for sequencing. The single stranded phagmids rescued with helper phage M13KO7 were sequenced using 0.5−1.0 pmol of both T-7 and exon-specific 5′- primer in separate reactions with sequenase V.2 (USB, USA). p53 exons cloned from normal peripheral blood DNA were used as control templates for sequencing. The sequencing reactions were run on a 6% polyacrylamide 7 M urea gel which was fixed in acetic acid, dried and exposed to Fuji xomat X-ray films for 18−24 h.

### Results

*Detection of p53 overexpression using flow cytometry*

Immunocytochemical analysis of the cell lines and breast tumour tissues clearly indicated that the antibodies PAb 1801 and DO-7 specifically recognised nuclear accumulation of mutated p53 protein (Figure 1). Similarly, the flow cytometric immuno-4 analysis of the three test cell lines confirmed the sensitivty of the immunofluorescence assay in which the percentage of cells overexpressing p53 protein was found to vary according to the corresponding genetic abnormality. Thus, both T-47D and SW 480 with known mutations in exon 6 and 9 respectively, had 80−90% of cells over-expressing the protein, whereas only 0.3% of HL-60 cells with a deleted p53 gene were found to express the protein (Figure 2). In addition, the specificity of the assay could be

**Figure 1** Nuclear accumluation of p53 in (**a**) SW480 cells stained with monoclonal antibody PAb1801 (10 $\mu$g ml$^{-1}$); (**b**) SW480 cells stained with non-specific antibody IgG1 against CD4 (10 $\mu$g ml$^{-1}$); (**c**) paraffin-embedded breast tumour stained with PAb1801 (20 $\mu$g ml$^{-1}$); (**d**) paraffin-embedded breast tumour stained with non-specific antibody IgG1 against CD4 (20 $\mu$g ml$^{-1}$). All photomicrographs were taken at ×400 magnification. (**e**) is a lower power (100×) H and E section of the tumour specimen shown in **c** and **d** under magnification.

increased by gating the immunofluorescence events on the basis of their DNA profile, thereby reducing the background fluorescence to a minimum. As expected p53 specific immunofluorescence could be seen confined to cells not only in $G_1$ and S, but also in $G_2$/M phases of the cell cycle owing to stabilisation of the mutant protein.

A total of 110 breast tumours were analysed for p53 expression. Forty (36%) tumours were found to have less than 5% p53-positive cells and were classified as immunonegative. Seventy (64%) tumours had more than 5% p53-positive cells and were considered immunopositive. Of these, 15 tumours had more than 15% p53-positive cells. These were classified as highly immunopositive.

### *PCR–SSCP analysis of p53 mutations in exons 5–8*

To avoid ambiguity of results, the 15 highly immunopositive tumours and 15 completely immunonegative tumours were analysed for p53 mutations at the genomic level by PCR–SSCP. We excluded tumours showing moderate or a b

**Figure 2** Immuno-4 analysis of the control cell lines (**a**) SW480 and (**b**) HL-60, by FCM. The program performs statistical analysis on two single parameter immunofluorescence histograms and normalises and overlays the control histogram on the test histogram in order to match best the analysis region. It then substracts the control histogram from the test histogram on channel-to-channel basis. The percentage positive fraction is calculated from the sum of differences for all channels.

low level expression of p53 because of possible misinterpretation of results arising from a dilution effect owing to the presence of a large number of normal cells in such tumours. The sensitivity of PCR–SSCP to resolve single base changes on non-denaturing polyacrylamide gel (PAGE) was checked with p53 exon 6 amplified from T-47D cell line known to carry a mutation in exon 6. Normal peripheral blood DNA amplified p53 exon 6 was run as the wild-type control. Mutation was identified by the presence of aberrantly migrating band(s) (Figure 3). Although the exon 6 mutation of T-47D could be resolved on PAGE, none of the breast tumours showed mobility shift for exon 6 on PAGE. These findings prompted us to try the mutation detection and enhancement (MDE) gel, which has been reported to enhance resolution of SSCP patterns, particularly of exon 6 (Soto and Sukumar, 1992). Two out of 15 tumours showed mobility shift under these conditions (Figure 3).

Overall, PCR–SSCP detected mutations in 40% (12 30) carcinomas. Eleven of these 12 mutations were in immunopositive tumours and one in immunonegative tumour. The distribution of these mutations was six in exon 5, two in exon 6, three in exon 7 and one in exon 8 (Figures 3 and 4).

### *Genomic sequencing of exons 5–8*

The p53 coding region was sequenced across exons 5–8 in all the 30 tumours selected for genomic analysis. Eleven tumours out of 30 (36.6%) were found to be mutated on sequencing. These mutations were present in all four exons analysed (Table I). Exon 5 showed mutation in five tumours, exon 6 in two, exon 7 in three and exon 8 in one tumour. The majority of these mutations were base substitution mutations (Figure 5). However, one tumour in exon 7 showed a frameshift mutation (Figure 5) and one immunonegative tumour had deletion of the p53 gene. The latter, therefore, could not be used for PCR–SSCP and sequencing. All the rest had wild-type sequence.

### *Correlation between immunochemical detection of p53, PCR–SSCP and p53 gene mutation*

Eleven out of 15 immunopositive tumours were found to have mutation by PCR–SSCP. Genomic sequencing confirmed the presence of mutation in ten of these 11 tumours. Thus, one immunopositive tumour that did show mutation in exon 5 by SSCP, however, failed to show mutation on sequencing. Since exon 5 had been amplified in two overlapping fragments, it is unlikely that we would have

**Figure 3** Electrophoretic pattern of p53 exon 6 DNA on SSCP analysis. Numbers represent breast tumours given in Table I. For details, refer to the Materials and methods and results sections.

**Figure 4** Electrophoretic pattern of p53 exon 5, exon 7 and exon 8 DNA amplified from human breast tumours (BTs) and normal human peripheral blood lymphocyte (NHPBL). BT-2, BT-3 and BT-4 show mobility shifts for exon 5. All the three tumours (BT-1, BT-6 and BT-8) show mobility shift for exon 7, whereas only BT-11 shows mobility shift for exon 8. Numbers represent breast tumours given in Table I. For details, refer to the Materials and results sections.

**Table I** Correlation between immoreactivity, mutation by PCR-SSCP and genomic sequencing of p53 gene in breast tumours

| *Breast tumour* | *Immuno (+) cells (%)* | *SSCP status* | *Base change* | *Codon change* |
|---|---|---|---|---|
| Immunopositive | | | | |
| BT-1 | 23.4 | Ex-7 | Deletion of T at codon 241 | Frameshift |
| BT-2 | 26.2 | Ex-5 | A→T | CAT→CTT |
| BT-3 | 31.4 | Ex-5 | A→T | CAC→CTC |
| BT-4 | 30.3 | Ex-5 | A→T | GAG→GTG |
| BT-5 | 18.0 | ND | ND | |
| BT-6 | 30.2 | Ex-7 | C→A | ATC→ATA |
| BT-7 | 30.9 | Ex-6 | C→G | CCC→CCG |
| BT-8 | 40.3 | Ex-7 | A→C | AAC→CAC |
| BT-9 | 34.8 | Ex-6 | G→C | TTG→TTC |
| BT-10 | 17.3 | Ex-5 | C→A | CAG→AAG |
| BT-11 | 21.7 | Ex-8 | T→G | GTG→GGG |
| BT-12 | 31.7 | Ex-5 | No mutation | |
| BT-13 | 30.0 | ND | ND | |
| BT-14 | 16.3 | ND | ND | |
| BT-15 | 14.8 | ND | ND | |
| Immunonegative | | | | |
| BT-16 | 4.6 | ND | ND | |
| BT-17 | 0.7 | ND | ND | |
| BT-18 | 0.3 | ND | ND | |
| BT-19 | 0.8 | EX-5 | 3-bp deletion and codon change | TCA→TCG<br>CAG→ACG |
| BT-20 | 1.8 | ND | ND | |
| BT-21 | 2.5 | ND | ND | |
| BT-22 | 1.0 | ND | ND | |
| BT-23 | 0.3 | ND | ND | |
| BT-24 | 1.9 | ND | ND | |
| BT-25 | 3.7 | ND | ND | |
| BT-26 | 2.4 | ND | ND | |
| BT-27 | 0.3 | Deletion | | |
| BT-28 | 0.6 | ND | ND | |
| BT-29 | 1.3 | ND | ND | |
| BT-30 | 2.5 | ND | ND | |

ND, not detected.

missed the mutation. Four immunopositive tumours failed to show mutation by both PCR-SSCP and genomic sequencing. Of the 15 immunonegative tumours, one tumour actually harboured a 3 bp deletion and change of codon at two consecutive positions. Additionally, one immunonegative tumour had undergone deletion of at least exons 5-8 of the p53 gene. Overall, immunoreactivity correlated with both PCR-SSCP and genomic sequencing in 80% of cases (24/30), whereas there was 96.5% concordance between PCR-SSCP and genomic sequencing.

### Discussion

Several assays have been used to analyse the presence of p53 mutations in human cancer, of which immunodetection of p53 protein and PCR-SSCP are the most frequently used methods. Both the methods are limited by their vulnerability to experimental conditions, resulting in difficult interpretation of the results. We have, therefore, compared systematically the immunochemical method with PCR-SSCP and genomic sequencing for p53 mutations in human breast tumours. Initially, 110 breast tumour were screened for p53 expression by dual parameter flow cytometry. A comparative analysis was then carried out in a subset of 15 highly immunopositive tumours and 15 completely immunonegative tumours. Dual parameter FCM analysis was preferred over immunocytochemistry for the reasons given below.

The specificity of the antibodies PAb 1801 and DO-7 to detect nuclear accumulation of p53 was confirmed by immunocytochemistry in cultured cell lines and by immunohistochemistry on formalin-fixed paraffin-embedded human breast tumour tissues. p53 expression was found confined to neoplastic epithelial areas, specifically recognising nuclear accumulation of p53 protein. It was consistent with the findings of Bartek *et al.* (1990*b*). However, it was observed that cultured cells without formalin fixation produced better staining patterns. Hence, we analysed p53 expression by dual parameter flow cytometry, which involved only mild fixation in paraformaldehyde. Moreover, the flow cytometric immunofluorescence assay used was found to be quantitative and highly specific as it monitors p53 expression and its DNA content simultaneously, thereby minimising background fluorescence. It also enabled visual confirmation of G1-S phase-specific p53 expression.

Sixty-four per cent of tumours were found to be immunopositive by the flow cytometric assay as against 20-57% reported in earlier studies (Cattriotie *et al.*, 1988; Bartek *et al.*, 1990; Davidoff *et al.*, 1991; Deng *et al.*, 1994). Several factors may have been responsible for the higher percentage of immunopositive tumours reported here. Firstly, the antibodies may recognise the epitopes of p53 more efficiently in its natural conformation under the conditions that we used. Secondly, in contrast to the earlier definitions of immunopositivity, such as: 'the presence of 20% immunopositive cells' (Isola *et al.*, 1992) or 'uniform p53

**Figure 5** Genomic sequencing of exons 5–8 of the p53 gene in human breast tumours. Each sequence is shown 5′ (bottom) to 3′ (top). Sequences are shown for BT-1, BT-6 (**b** and **c**) showing mutation in exon 7 and BT-3 (**a**) showing mutation in exon 5. Numbers refer to the breast tumours given in Table I.

staining in all the cells' (Davidoff *et al.*, 1991), we have uesd the presence of 5% p53-positive cells as a cut-off to distinguish immunopositive from immunonegative tumours.

Immunoreactivity correlated with p53 mutations detected by SSCP and sequencing in 80% (24/30) cases. Four out of 15 immunopositive tumours did not show mutation. Contrariwise, 1/15 immunonegative tumours were found to have mutation by SSCP and sequencing. One tumour had a deleted p53 gene. The four immunopositive tumours that failed to show mutation had 15%, 16%, 18% and 30% p53-positive cells respectively. It may be argued that mutations were diluted beyond the detection limit of SSCP technique and, therefore, remained undetected in the first three of these tumours, or that the threshold of immunopositivity chosen by us (>15%) is still within the range plausibly caused by variable induction of wild-type p53 in response to DNA damage/genome instability. But the lack of mutation in the immunopositive tumour with as high as 30% p53-positive cells, indicates that these tumours may be expressing the wild-type protein. This was further confirmed by sequencing. The immunopositive phenotype may have been due to its stabilisation on its interaction with cellular proteins such as hsp-70 or some unknown viral proteins. Moll *et al.* (1995) have suggested that cytoplasmic sequestration of wild-type p53 may be another mechanism for its inactivation, although this issue cannot be resolved in our study, since we determined p53 expression by flow cytometry and not by immunohistochemistry. Of course, the possibility of these tumours harbouring a mutation outside the conserved exons 5–8 cannot, however, be ruled out.

Point missense mutations were observed in the sequence-specific DNA binding domain of p53 in 11 tumours. They occured at highly conserved residues (codons 143, 166, 167, 168, 171, 179, 206, 219, 238, 241, 250 and 271). As p53 is a DNA binding protein, mutation at codon 179 may have drastic consequences as the amino acid histidine at this position is involved in zinc coordination and DNA binding (Cho *et al.*, 1994). Similarly, all the other mutations found also lie in the $L_2$ loop of the $\beta$-sandwich (163–195 residues), which is involved in maintaining the tetrahedral geometry of zinc. Codon 143 is directly involved in establishing DNA contact (Cho *et al.*, 1994). It, therefore, might be clinically important to determine the difference in behaviour of tumours between those having mutations at these functionally critical residues and those with mutations at other sites.

The largest number of mutations were found in exon 5 in agreement with others (Osborne *et al.*, 1991; Deng *et al.*, 1994). Most of these mutations were transversions, where purine and pyrimidine residues were interchanged. But, as opposed to a higher frequency (71%) of G→C transversions found by Deng *et al.* (1994), more A→T transversions (60%) were observed in our study. Interestingly, one tumour which was immunonegative and had shown mutation in exon 5 by SSCP on genomic sequencing was found to have undergone a 3 bp deletion and change of codons at two consecutive positions. The mutations might have altered the protein conformation such that the antibody no longer recognised the protein.

Deletion of p53 is a relatively rare event (8.1%) (for review see Levine *et al.*, 1994). In the present study, one out of the 30 tumours was found to have undergone deletion at least within the conserved exons 5–8. None of these exons could be amplified from this sample. Yet when DQ-$\alpha$ locus-specific primers were used to check if other loci from the sample could be amplified, a DNA fragment specific to that locus was invariably obtained. The deletion of the p53 gene may be responsible for the immunonegative phenotype of this tumour.

In conclusion, the study indicates that there is 80% concordance among the three methods to detect p53 mutation. PCR–SSCP and genomic sequencing correlated with each other in 96.5% of cases. Immunochemical detection of p53 was, therefore, not an absolute indicator of p53 gene mutation. Since it is the genomic mutation of p53 which confers growth advantage to the affected cell, the ultimate aim of any p53 assay should be to detect a genomic defect. Detection of overexpression of p53 protein alone (in the absence of p53 mutation) may be misleading, as stabilisation of p53 may be a transient phenomenon and can revert to normality with time.

### References

ALLRED DC, CLARK GM, ELLEDGE R, FUQUA SA, BROWN RW, CHAMNESS GC, OSBORNE CK AND MCGUIRE WL. (1993). Association of p53 protein expression with tumour cell proliferation rate and clinical outcome in node negative breast cancer. *J. Natl Cancer Inst.*, **85,** 200–206.

BANKS L, MATLASHEWSKI G AND CRAWFORD L. (1986). Isolation of human p53 specific monoclonal antibodies and their use in the studies of human p53 expression. *Eur. J. Biochem.*, **159,** 529–534.

BARTEK J, BARTKOVA J, VOJTSEK B, STASKOVA Z, REJTHAR A, KOVARIK J AND LANE DP. (1990*b*). Patterns of expression of the p53 tumour suppressor in human breast tissues and tumours *in situ* and *in vitro*. *Int. J. Cancer*, **46**, 839–844.

BARTEK J, IGGO R, GANNON J AND LANE DP. (1990*a*). Genetic and immunochemical analysis of mutant p53 in human breast cancer cell lines. *Oncogene*, **5**, 893–899.

CATTORETTI G, RILKE F, ANDREOLAR S, D'AMATO L AND DELIA D. (1988). p53 expression in breast cancer. *Int. J. Cancer*, **41**, 178–183,

CHO Y, GORIA S, JEFFREY PD AND PAVLETICH NP. (1994). Crystal structure of a p53 tumour suppressor–DNA complex: understanding tumorigenic mutations. *Science*, **265**, 346–355.

DAVIDOFF AM, HUMPHREY PA, IGLEHART JD AND MARKS JR. (1991). Genetic basis for p53 overexpression in human breast cancer. *Proc. Natl Acad. Sci. USA*, **88**, 5006–5010.

DENG G, CHEN LC, SCHOTT DR, THOR A, BHARGAVA V, LJUNG BM, CHEW K AND SMITH HS. (1994). Loss of heterozygosity and p53 gene mutations in breast cancer. *Cancer Res.*, **54**, 499–505.

GANNON JV, GREAVES R, IGGO R AND LANE DP. (1990). Activating mutations in p53 produce a common conformational effect. A monoclonal antibody specific for the mutant form. *EMBO J.*, **9**, 1595–1602.

HALL PA AND LANE DP. (1994). p53 in tumour pathology: Can we trust immunohistochemistry? – Revisited! *J. Pathol.*, **172**, 1–4.

HALL PA, MCKEE PH, MENAGE HD, DOVER R AND LANE DP. (1993). High levels of p53 protein in UV irradiated human skin. *Oncogene*, **8**, 203–207.

HOLLSTEIN M, SIDRANSKY D, VOGELSTEIN B AND HARRIS CC. (1991). p53 mutations in human cancers. *Science*, **253**, 49–53.

IGGO R, GATTER K, BARTEK J, LANE D AND HARRIS AL. (1990). Increased expression of mutant forms of p53 oncogene in primary lung cancer. *Lancet*, **335**, 675–679.

ISOLA J, VISAKORPI T, HOLLI K AND KALLIONIEMI OP. (1992). Association of overexpression of tumour suppressor protein p53 with rapid cell proliferation and poor prognosis in node negative breast cancer patients. *J. Natl Cancer Inst.*, **84**, 1109–1114.

KOHLER MF, NISHI H, HUMPHREY PA, SARKI H, MARKS J, BAST RC, CLARKE PEARSON DL, BOYD J AND BERCHUCK A. (1993). Mutation of the p53 tumour suppressor gene is not a feature of endometrial hyperplasia. *Am. J. Obstet. Gynecol.*, **169**, 690–694.

LANE DP. (1994). The regulation of p53 function: Steiner award lecture. *Int. J. Cancer*, **57**, 623–627.

LEVINE AJ, PERRY ME, CHANG A, SILVER A, DITTMER D, WU M AND WELSH D. (1994). The 1993 Walter Hubert Lecture: the role of the p53 tumour suppressor gene in tumorigenesis. *Br. J. Cancer*, **69**, 409–416.

MANIATIS T, FRITSCH J AND SAMBROOK J. (eds). (1982). *Molecular Cloning: a Laboratory Manual.* Cold Spring Harbour Laboratory Press: Cold Spring Harbour, NY.

MATELASHEWSKI G, BANKS L, PIM D AND CRAWFORD L. (1986). Analysis of human p53 protein and mRNA levels in normal and transformed cells. *Eur. J. Biochem.*, **154**, 665–672.

MILNER J AND MEDCALF EA. (1991). Cotranslation of activated mutant p53 with wild type drives the wild type p53 into the mutant conformation. *Cell*, **65**, 765–774.

MOLL UM, LAQUAGLIA M, BENARD J AND RIOU G. (1995). Wild type p53 protein undergoes cytoplasmic sequesteration in undifferentiated neuroblastomas but not in differentiated tumours. *Proc. Natl Acad. Sci. USA*, **92**, 4407–4411.

NAVONE NM, TRONCOSO P, PISTERS LL, GOODROW TL, PALMER JL, NICHOLS WW, ESCHENBACH ACV AND CONTI CJ. (1993). p53 protein accumulation and gene mutation in the progression of human prostate carcinoma. *J. Natl Cancer Inst.*, **85**, 1657–1669.

NIGRO JM, BAKER SJ, PREISINGER AC, JESSUP JM, HOSTETTER R, CLEARY K, BIGNER SH, DAVIDSON N, BAYLIN S, DEVILEE P, GLOVER T, COLLINS FS, WESTON A, MODALI R, HARRIS CC AND VOGELSTEIN B. (1989). Mutations in p53 gene occur in diverse human tumour types. *Nature*, **342**, 705–708.

ORITA S, SUZUKI Y, SEKIYA T AND HAYASHI K. (1989). Rapid and sensitive detection of point mutations and DNA polymorphisms using the polymerase chain reaction. *Genomics*, **5**, 874–879.

OSBORNE RJ, MERLO GR, VENESIO MT, LISCIA VDS, CAPPA APM, TAKAHASHI CT, NAU MM, CALLAHAN R AND MINNA JD. (1991). Mutations in the p53 gene in primary human breast cancers. *Cancer Res.*, **51**, 6194–6198.

RASBRIDGE SA, GILLET CE, SEYMOUR AM AND MILLIS RR. (1993). The effect of chemotherapy on histological and biological features of breast carcinoma. *J. Pathol.*, **169** (suppl.), 191.

RODRIGUES NR, ROWAN A, SMITH ME, KERR IB, BODOMER WF, GANNON J AND LANE DP. (1990). p53 mutations in colorectal cancer. *Proc. Natl Acad. Sci. USA*, **87**, 7555–7559.

SOTO D AND SUKUMAR S. (1992). Improved detection of mutations in the p53 gene in human tumours as single stranded conformation polymorphs and double stranded heteroduplex DNA. *PCR Methods Applications*, **2**, 96–98.

THOR AD, MOORE DH, EDGERTON SM, KAWASAKI ES, REIHSOUS E, LYNCH HT, MAARCUS JN, SCHWARTZ L, CHEN LC, MAYALL BH AND SMITH HS. (1992). Accumulation of p53 tumour suppressor gene protein: an independnet marker of prognosis in breast cancer. *J. Natl Cancer Inst.*, **84**, 845–855.

TOGUCHIDA J, YAMAGUCHI T, DAYTON SH, BEAUCHAMP RL, HERRERA GE ISHIZAKI K, YAMAMURO T, MEYERS PA, LITTLE JB, SASAKI MS, WEICHSEL BAUM RR AND YANDELL DW. (1992). Prevalence and spectrum of germline mutations of the p53 gene among patients with sarcoma. *N. Engl. J. Med.*, **326**, 1301–1308.

WYNFORD-THOMAS D. (1992). p53 in tumour patholoogy: can we trust immunocytochemistry? *J. Pathol.*, **166**, 329–330.